# Sex differences in the impact of the Mediterranean diet on systemic inflammation

**DOI:** 10.1186/s12937-015-0035-y

**Published:** 2015-05-12

**Authors:** Alexandra Bédard, Benoît Lamarche, Louise Corneau, Sylvie Dodin, Simone Lemieux

**Affiliations:** 1 Institute of Nutrition and Functional Foods (INAF), 2440 Hochelaga Boulevard, Laval University, Québec, Qc G1V 0A6 Canada; 2 School of Nutrition, Pavillon Paul-Comtois, 2425 rue de l’Agriculture, Laval University, Québec, Qc G1V 0A6 Canada; 3 Department of Obstetrics and Gynaecology, Pavillon Ferdinand-Vandry, 1050 Medicine Avenue, Laval University, Québec, Qc G1V 0A6 Canada

**Keywords:** Sex, Mediterranean diet, C-reactive protein, Men, Women

## Abstract

**Background:**

Some intervention trials have reported a reduction in systemic inflammation with the Mediterranean diet (MedDiet) while others have observed no effect. Despite the fact that sex differences have been highlighted in the inflammatory regulation, it is still not known whether MedDiet exerts similar effects on systemic inflammation in men and women. The aim of this study was therefore to investigate sex differences in the effects of the MedDiet on high-sensitivity C-reactive protein (hs-CRP).

**Findings:**

Participants were 35 men and 27 premenopausal women (24–53 years) presenting a slightly deteriorated lipid profile. All foods were provided to participants during a 4-week isocaloric MedDiet. At baseline, women had higher hs-CRP concentrations than men (P = 0.03). No sex difference was observed in hs-CRP response to the MedDiet (P for sex-by-time interaction = 0.36), with both men and women experiencing no change (respectively P = 0.62 and P > 0.99). When subgroups were formed according to hs-CRP concentration before the MedDiet phase, men with elevated baseline values (≥2 mg/l) experienced a reduction in hs-CRP over time with the MedDiet (−26.5 %) while an increase was observed in men with lower baseline values (+96.6 %; P for group-by-time interaction = 0.02). This pattern of change was not observed in women.

**Conclusions:**

Results from this controlled feeding study suggest that men and women have similar effects from the MedDiet on systemic inflammation. The individual’s overall inflammatory status seems to influence these effects, but only in men.

**Trial registration:**

This clinical trial was registered at www.clinicaltrials.gov as NCT01293344.

## Findings

### Introduction

The implication of low-grade, chronic inflammation in the formation, progression and rupture of atherosclerotic plaques is now well-recognized [1]. Accordingly, elevated C-reactive protein (CRP), a marker reflecting the individual’s systemic inflammatory status, has been consistently associated with increased risk of coronary heart disease events [2, 3] and type 2 diabetes [4]. There is growing evidence that adopting the traditional Mediterranean diet (MedDiet) reduces systemic inflammation [5]. The MedDiet is characterized by an abundance of plant-based foods, such as fruits, vegetables, whole grain cereals, nuts and legumes; olive oil as the main source of fat; moderate amounts of fish, poultry, dairy products and eggs; relatively low amounts of red meat and sweets and moderate amounts of red wine with meals [6]. However, even if most of the intervention trials have reported that this food pattern reduces CRP concentrations [7–11], some have observed no effects [12–15]. Accordingly, the investigation of factors that may influence the anti-inflammatory effects of this healthy food pattern is of great interest. Sex has been highlighted as a determinant of the inflammatory regulation. In fact women are characterized by a higher inflammatory overall burden than men [16]. Also, effects of sex hormones on inflammatory status have been documented, estrogens being now recognized for their anti-inflammatory properties in women [17]. However it is still not known whether MedDiet exerts the same effect on inflammation in men and women. The aim of this study was therefore to investigate sex differences in the effects of the MedDiet on high-sensitivity C-reactive protein (hs-CRP) concentrations. As estrogens have anti-inflammatory properties in premenopausal women [17], and that the MedDiet has been previously shown to reduce estrogen concentrations in women [18], we hypothesized that premenopausal women benefit less from the anti-inflammatory effects of the MedDiet than men.

## Methods

### Participants

Thirty-eight men and 32 premenopausal women (24–53 years) took part of this study. The main inclusion criteria were to have a slightly elevated low-density lipoprotein cholesterol (LDL-C) concentrations (between 3.4 and 4.9 mmol/l) or total cholesterol to high-density lipoprotein cholesterol (HDL-C) ratio ≥5.0, and at least one of the four following cardiovascular disease (CVD) risk factors: waist circumference >94 cm in men and >80 cm in women; triacylglycerol (TAG) concentration ≥1.7 mmol/l; fasting glycemia between 6.1 and 6.9 mmol/l and/or blood pressure levels ≥130/85 mm Hg. More details about inclusion and exclusion criteria have been reported elsewhere [19]. Women using systemic hormonal contraceptives were excluded. All subjects signed an informed consent form before their inclusion in the study, which has been approved by the Laval University Research Ethics Committee. Power analysis indicated that a total sample size of n = 62 is sufficient to detect significant changes in hs-CRP concentrations (repeated measures, within-between interaction) with a small effect-size estimate (Cohen’s d of 0.20), and with an α = 0.05 and a power (1-β error probability) of 0.95 (G*Power Version 3.0.10, Franz Faul, Universität Kiel, Germany).

### Study design

The study protocol consisted in a 4-week run-in period, immediately followed by a 4-week fully-controlled MedDiet phase. Firstly, during the 4-week run-in period, participants had to comply with the recommendations of the Canada’s Food Guide [20] as prescribed by a registered dietician. The purpose of this run-in period was to ensure similar dietary habits between men and women prior the controlled MedDiet phase, a goal that has been reached as previously reported [19]. Briefly, Canada’s Food Guide is an educational tool which promotes healthy eating for Canadians in order to reduce the risk of many chronic diseases and to achieve overall health and vitality. It indicates the recommended number of food guide servings per day for each of the four food groups (vegetables and fruits, grain products, milk and alternatives, and meat and alternatives) according to the age and sex of individuals.

Thereafter, during a 4-week fully-controlled feeding phase, subjects consumed an experimental MedDiet formulated to be concordant with the characteristics of the traditional MedDiet [6]. Details about the composition of the MedDiet are given in Table 1 and Table 2, as previously reported in other publications [19, 21]. Subjects were instructed to consume only the foods and beverages provided to them, which corresponded to 100 % of their estimated energy needs. More precisely, energy needs were estimated by averaging the energy requirements estimated by a validated FFQ [22] administrated at the beginning of the run-in period and energy needs as determined by the Harris–Benedict formula. Body weight was measured on weekdays just before lunch and in case of body weight variation, energy intake was modified. The amount of each food/drink provided to each participant during the MedDiet was proportional to his/her estimated energy needs. In order to evaluate compliance, participants were asked to note on a checklist foods consumed and, if needed, the amount of foods not consumed for each day of the controlled MedDiet phase. The overall compliance calculated from the food checklist in men and women was respectively 97.9 ± 3.6 % and 97.6 ± 3.2 %. Since sex hormones may influence the inflammatory status [17], women’s feeding was shortened or prolonged if needed in order to be able to carry out all tests in the early follicular phase of their menstrual cycle (mean duration of the feeding period in women 28.8 ± 4.3 days).

**Table 1:** Servings of key foods of the Mediterranean pyramid consumed daily during the experimental Mediterranean diet phase for a 10 460 kJ/d (2500 kcal/d) menu

Key foods^a^	MedDiet (servings/d)
Olive oil (ml)	43.3
Whole grains products	5.7
Fruits and Vegetables	16.1
Legumes	0.5
Nuts	0.9
Cheese and yogurt	2.0
Fish	1.3
Poultry	0.9
Eggs	0.3
Sweets	0.3
Red meat	0.2
Red wine	1.3

*MedDiet* Mediterranean diet

^a^Extra virgin and virgin olive oils were used. Serving size for whole grains products = 125 ml (rice, pasta, bulgur, couscous), one bread piece or 30 g cereal; Serving size for fruits and vegetables = 125 ml; Serving size for legumes = 175 ml and for nuts = 30 g; Serving size for fish, poultry and red meat = 75 g; Serving size for egg = 100 g; Serving size for dairy products (mostly low fat cheese and yogurt) = 50 g cheese, 175 g yogurt and 250 ml milk; Serving size for red wine = 150 ml

This table has been previously published in other publications [19, 21]

**Table 2:** Daily nutritional composition of the experimental Mediterranean diet for a 10 460 kJ/d (2500 kcal/d) menu

	MedDiet
	For 10 460 kJ/d (2500 kcal/d)
Energy (kJ)	10 460
Carbohydrate (% of total energy)	46.0
Fiber (g)	42.3
Protein (% of total energy)	17.0
Fat (% of total energy)	32.0
SFA (% of total energy)	6.7
MUFA (% of total energy)	18.1
PUFA (% of total energy)	4.7
Cholesterol (mg)	289.7
Alcohol (% of total energy)	5.0
MUFA to SFA ratio	2.7
Sodium (mg)	3039

*MedDiet* Mediterranean diet

This table has been previously published in other publications [19, 21]

### CRP measurements

Fasting blood samples were collected after the run-in period (*i.e.* just before the controlled MedDiet phase, referred as baseline values) and immediately after the MedDiet. Serum concentrations of hs-CRP were measured using a high-sensitivity enzyme immunoassay test kit (BioCheck Inc., Foster City, CA; coefficients of variation: intra-assay ≤ 7.5 %, inter-assay ≤ 4.1 %).

### Statistical analysis

Statistical analyses were performed with the SAS statistical package version 9.4 (SAS Institute Inc., Cary, NC, USA). Time and sex-by-time interaction effects on hs-CRP concentrations were assessed by using MIXED procedures for repeated measurements followed by Tukey-Kramer tests. Participants with hs-CRP concentrations greater than 10 mg/l (indicative of an acute inflammation process [23]) before or after the MedDiet phase were excluded from our analyses (three men and five women). A P ≤ 0.05 was considered as statistically significant.

## Results

At baseline, men and women had similar mean age and body mass index (BMI) (Table 3). However, men were characterized by higher body weight and waist circumference, and displayed higher values for TAG, total cholesterol/HDL-C ratio, systolic and diastolic blood pressures and fasting glucose, and a lower value for HDL-cholesterol than women (Table 3). The degree of concordance of the diet with the traditional MedDiet, as assessed by the Mediterranean score after the run-in phase based on the Canada’s Food Guide [24], was similar in men and women (Table 3).

**Table 3:** Characteristics of men and women at baseline^a^

	Men (n = 35)		Women (n = 27)		Sex difference^b^
	Mean	SD	Mean	SD	*P*-value
Age (years)	43.0	7.2	41.4	7.3	0.3928
Body weight (kg)^c^	92.1	14.1	74.9	9.7	<0.0001
BMI (kg/m^2^)^c^	29.2	3.2	28.4	3.2	0.2881
Waist circumference (cm)^c^	102.7	11.0	94.7	8.1	0.0018
TAG (mmol/l)^c^	1.86	1.19	1.34	0.65	0.0273
LDL-cholesterol (mmol/l)	3.65	0.72	3.56	0.51	0.5896
HDL-cholesterol (mmol/l)^c^	1.12	0.30	1.33	0.25	0.0020
Total cholesterol/HDL-C	5.24	1.03	4.25	0.77	<0.0001
Systolic blood pressure (mm Hg)	117.3	12.9	107.2	10.2	0.0015
Diastolic blood pressure (mm Hg)	80.4	9.2	72.1	8.0	0.0005
Fasting glucose (mmol/l)	5.87	0.37	5.54	0.44	0.0019
Mediterranean score (arbitrary units)^d^	25.1	6.0	24.5	4.8	0.6541

*SD* standard deviation, *BMI* body mass index, *TAG* triacylglycerol, *LDL* low-density lipoprotein, *HDL* high-density lipoprotein

^a^These characteristics were measured after the run-in period, *i.e.* immediately before the controlled MedDiet phase

^b^Sex differences were determined using the Student’s *t*-test for unpaired data, except for age for which Wilcoxon-Mann–Whitney test was used

^c^Analysis was performed on transformed values

^d^From 0 to 44 points, a score of 44 implies a food pattern which is perfectly concordant with the traditional MedDiet

**Fig. 1:**
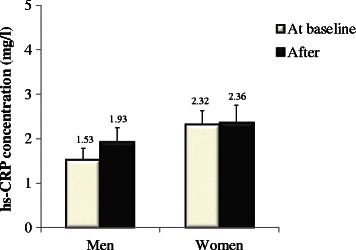
hs-CRP concentrations observed in men (left, n = 35) and women (right, n = 27) at baseline and after the 4-week Mediterranean diet. MIXED procedures for repeated measurements followed by Tukey-Kramer tests were used. Data are means ± SEM

At baseline, women had higher hs-CRP concentrations than men (1.53 ± 1.49 mg/l for men and 2.32 ± 1.63 mg/l for women; P for sex difference = 0.03). No change was observed for hs-CRP concentrations over time with the MedDiet in both men and women (respectively P = 0.62 and P > 0.99, P for sex-by-time interaction = 0.36; Fig. 1).

**Fig. 2:**
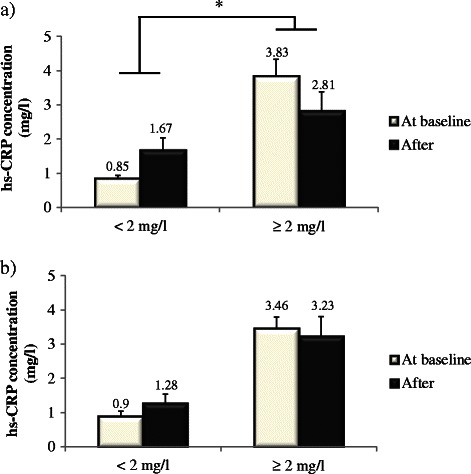
hs-CRP concentrations observed in men (**a**) and women (**b**) at baseline and after the 4-week Mediterranean diet according to their hs-CRP baseline value. MIXED procedures for repeated measurements followed by Tukey-Kramer tests were used. *A group-by-time interaction was found in men (P = 0.02) but not in women (P = 0.11). Men with hs-CRP < 2 mg/l, n = 27; men with hs-CRP ≥ 2 mg/l, n = 8; women with hs-CRP < 2 mg/l, n = 12; women with hs-CRP ≥ 2 mg/l, n = 15. Data are means ± SEM

When subgroups were formed according to hs-CRP concentrations before the MedDiet phase, men with elevated baseline values (≥2 mg/l, as defined in the Canadian guidelines for the diagnosis and treatment of dyslipidemia and prevention of CVD in the adult [25, 26]) experienced a reduction in hs-CRP over time with the MedDiet (−26.5 %) while an increase was observed in men with lower baseline values (<2 mg/l, +96.6 %; P for group-by-time interaction among men = 0.02; Fig. 2a). This pattern of change was not observed in women (P for group-by-time interaction among women = 0.11; Fig. 2b).

Adjustments for the small but significant weight loss during the MedDiet phase (−1.2 kg or 1.3 % of initial body weight in men, P < 0.0001 and −0.6 kg or 0.7 % in women, P = 0.04; P for sex-by-time interaction = 0.09) did not influence results obtained (not shown). Changes in body weight during the controlled MedDiet were not associated with changes in hs-CRP concentrations (r = −0.02, P = 0.89 in men and r = −0.13, P = 0.52 in women). No change was observed for waist circumference in both sexes.

## Discussion

Results from this fully-controlled feeding study suggest that men and women have similar effects from the MedDiet on systemic inflammation. In fact, in this sample of individuals characterized by moderately elevated CRP concentrations, no beneficial effects from the MedDiet were observed, irrespective of the sex. However, results suggest that the variability in the anti-inflammatory effects of the MedDiet might be attributed in part to the individual’s overall inflammatory status but this observation seems to be more specific to men.

Despite the fact that men and women differ substantially with respect to inflammatory regulation, very limited data exist on sex differences in the impact of diet on inflammatory status. In the case of the MedDiet, an observational study has reported reduced CRP concentrations in men, but not in women, who consumed a diet more closely in accordance with the MedDiet [27] while another study observed this association irrespective of the sex [28]. For interventional trials, only the Prevención con Dieta Mediterránea (PREDIMED) study has previously documented the effects of the MedDiet on inflammatory status taking into account the sex [11]. Their study, consisting of a nutritional intervention among 772 high-risk individuals, indicates that the adherence to an energy-unrestricted MedDiet supplemented with extra-virgin olive oil reduces CRP concentrations compared with a low-fat diet, with subgroup analyses showing no difference between sexes. Results from our feeding study are therefore partly in line with those from the PREDIMED study, suggesting that the MedDiet has similar effect on inflammation in men and women.

However, our results are in disagreement with a meta-analysis of randomized controlled trials published in 2014 [5] which, as the PREDIMED study, reported a reduction of CRP concentrations with the MedDiet. However, further investigations of trials included in the meta-analysis highlight conflicting results between studies, some reporting a reduction of systemic inflammation with the MedDiet while almost half of the studies observed no significant effects. In an effort to improve our understanding of factors responsible for this divergence between studies, additional analyses from the present study suggest that the variability in the inflammatory response to the MedDiet might be attributed in part to the individual’s systemic inflammatory status, *i.e.* those with elevated CRP concentrations having anti-inflammatory effects from the MedDiet while those with low baseline concentrations experiencing a clinically non-significant increase in response to this food pattern. However this pattern of change was more specific to men, suggesting that sex may modulate to a certain extent the impact of the MedDiet on the inflammatory status.

It is important to consider that participants included in the present study were characterized by healthy dietary habits at baseline. In fact, participants had to comply with the recommendations of the Canada’s Food Guide during the four weeks preceding the controlled MedDiet phase [19]. It is therefore possible that changes in hs-CRP have started during this run-in phase, limiting subsequent changes during the controlled MedDiet phase. Therefore, results from the present study should not be over-interpreted and they suggest that, compared with the Canada’s Food Guide recommendations, the MedDiet has no further impact on hs-CRP concentrations.

The small body weight loss observed during the MedDiet phase may be view as a limitation. However, several studies have demonstrated that body weight loss is the best nonpharmacologic modality to reduce inflammation [29], which is in contrast with the nonsingnificant increase in hs-CRP concentrations observed in the present study. In addition, some studies have highlighted that a weight loss of at least 10 % is needed to have a significant effect on the inflammatory markers in overweight and obese individuals [30]. Moreover, additional analyses showed that the adjustment for body weight changes did not influence any of the results obtained. Therefore, these observations suggest that body weight loss observed in the present study was not a major limitation.

## Conclusions

Results from this feeding study suggest that the MedDiet exerts similar effects on inflammation in men and women. In addition, these results suggest that the variability in the anti-inflammatory effects of the MedDiet might be attributed in part to the individual’s overall inflammatory status; however this observation seems to be more specific to men. Additional clinical studies including several inflammatory markers and a larger sample size are of importance to further document the impact of sex on the inflammatory response to the MedDiet.

## Abbreviations

CRP: C-reactive protein
MedDiet: Mediterranean diet
hs-CRP: High-sensitivity C-reactive protein
LDL-C: Low-density lipoprotein cholesterol
HDL-C: High-density lipoprotein cholesterol
TAG: Triacylglycerol
FFQ: Food frequency questionnaire
BMI: Body mass index
SD: Standard deviation
